# A Rare Encounter: Unstable Vasospastic Angina Induced by Thyrotoxicosis

**DOI:** 10.3390/jcm13113130

**Published:** 2024-05-27

**Authors:** Artiomas Širvys, Arvydas Baranauskas, Povilas Budrys

**Affiliations:** 1 Clinic of Cardiac and Vascular Diseases, Faculty of Medicine, Vilnius University, 03101 Vilnius, Lithuania; arvydas.baranauskas@santa.lt (A.B.); povilas.budrys@santa.lt (P.B.); 2 Cardiology and Angiology Center, Vilnius University Hospital Santaros Klinikos, 08410 Vilnius, Lithuania

**Keywords:** unstable angina, coronary artery vasospasm, coronary angiography, intravascular ultrasound

## Abstract

Coronary artery vasospasm plays a crucial role in the prevalence of unstable angina. Despite common misdiagnosis, there is limited evidence on this topic. Here, we present a rare case of unstable vasospastic angina in a female with severe thyrotoxicosis. **Case Report**: A 62-year-old female patient was admitted to the cardiac intensive care unit due to crushing chest pain at rest. The patient exhibited ischemic changes on the ECG with a normal troponin I level. Recurrent chest pain prompted urgent coronary angiography, revealing generalized vasospasm of all coronary artery branches including the left main coronary artery. Intracoronary nitroglycerin injection partially alleviated the vasospasm; however, there was a persistent stenosis in the left main artery. Subsequent intravascular ultrasound demonstrated an anatomically normal left main artery. Post-procedure, laboratory tests revealed undetectable levels of thyroid-stimulating hormone and thyroid hormones above the detectable level. The patient was initiated on methimazole and discharged symptom-free, expecting a good prognosis under conservative management. **Conclusions**: Clinically significant coronary vasospasm triggered by thyrotoxicosis remains a rarity in clinical practice, often posing diagnostic challenges. This case emphasizes the significance of intracoronary nitroglycerin and intravascular ultrasound in discerning the etiology of coronary lesions seen on angiography. We advocate for these techniques to optimize invasive coronary artery diagnostics, enabling the selection of the appropriate treatment strategies and improving long-term prognosis.

## 1. Introduction

Acute coronary syndromes (ACSs) stand as a significant contributor to mortality rates across Europe [1]. Unstable angina (UA), characterized by myocardial ischemia at rest accompanied by typical chest pain and shortness of breath, is one of the key components of ACS [1]. While atherosclerotic disease of coronary arteries is widely acknowledged as the primary culprit behind ACS [2], it is essential to recognize that the vasospasm of coronary arteries can also provoke such episodes [3]. Vasospasm entails either focal or diffuse coronary arteries constriction, leading to severe stenosis and diminished blood flow to the myocardium, though it is deemed a functional rather than structural anomaly [4]. The incidence of coronary vasospasm is highest in individuals aged between 40 and 70 [5]. Also, the prevalence is higher among the Asian population than the European population [4,5]. General risk factors of coronary vasospasm are older age, cigarette smoking and higher levels of high-sensitivity C-reactive protein [6]. Notably, smoking has a stronger association with coronary vasospasm in younger patients [7]. Vasospasm may occur due to several factors, such as increased sympathetic activity, endothelial dysfunction, oxidative stress, smooth muscle cell hypercontractility and genetic factors [5]. Interestingly, some of these pathophysiological disturbances may be exacerbated by thyrotoxicosis, which is marked by elevated thyroid hormone activity due to the excessive production and concentration in the bloodstream [8]. The prevalence of hyperthyroidism and thyrotoxicosis in Western population stands at 1.2%, and Grave’s disease is the most common cause [8]. Akin to vasospastic angina, thyrotoxicosis is more common among females [4,8]. Clinically significant coronary vasospasm triggered by thyrotoxicosis remains a rarity in clinical practice, and it often poses diagnostic challenges [9]. Despite its significance and the prevalence of misdiagnoses, evidence on this topic remains scant, and it is largely limited to case reports and retrospective studies. Here, we present a rare case of unstable vasospastic angina in a middle-aged female with severe thyrotoxicosis.

## 2. Case Report

The case illustrates a rare manifestation of unstable angina attributed to a vasospasm in a 62-year-old woman. Her condition began to deteriorate about a week prior to hospitalization with the typical stress-induced chest pain escalating to discomfort even at rest. Managing her symptoms with orally administered isosorbide mononitrate (ISMN) (60 mg) proved effective, as the pain responded well to vasodilating medications. A few days before her admission, the patient noticed swelling in her ankles for the first time. After consulting a cardiologist at an outpatient clinic and observing signs of heart failure, she was referred to the internal medicine department at a secondary care hospital. An echocardiogram revealed preserved ejection fraction (EF of 50%), mild aortic valve stenosis and mild left ventricular hypertrophy. Given the high suspicion of unstable angina, the patient was promptly directed to a percutaneous coronary intervention (PCI) center. The patient had a medical history of arterial hypertension and dyslipidemia. 

Before admission to the secondary care hospital, the patient was prescribed Verapamil (180 mg), Trandolapril (2 mg) once daily, Atorvastatin (10 mg) once daily, and Isosorbide Mononitrate (20 mg) once daily. The treatment plan implemented at the secondary care hospital is outlined in Table 1.

**Figure 1:**
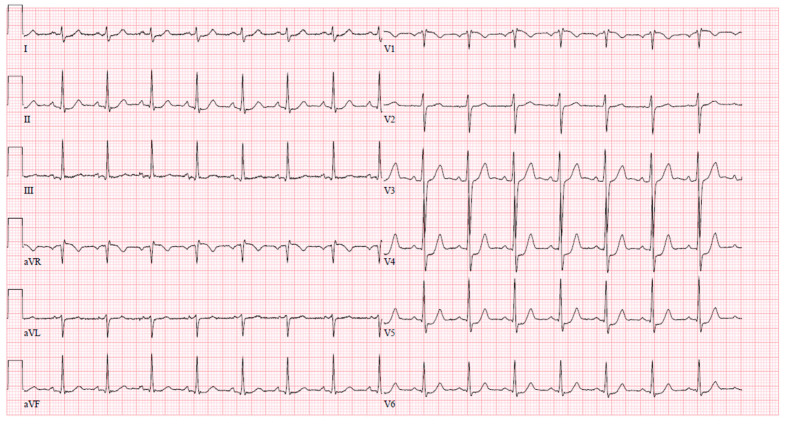
An ECG upon arrival at the cardiac ICU.

Upon arrival at the PCI center, the patient was admitted to the cardiac intensive care unit reporting severe chest pain spreading to the left arm, which was accompanied by dizziness and difficulty breathing. The initial electrocardiogram (ECG) upon admission revealed sinus tachycardia with evident ST segment depression in leads I, aVL, V4–6, and ST segment elevation in lead aVR (Figure 1). Despite this presentation, the high sensitivity troponin I level remained within normal limits at 12 ng/L (normal range ≤ 16 ng/L), leading to the patient being managed under the diagnosis of unstable angina. The medications prescribed during the patient’s stay in the PCI center’s intensive care unit (ICU) are detailed in Table 2.

**Figure 2:**
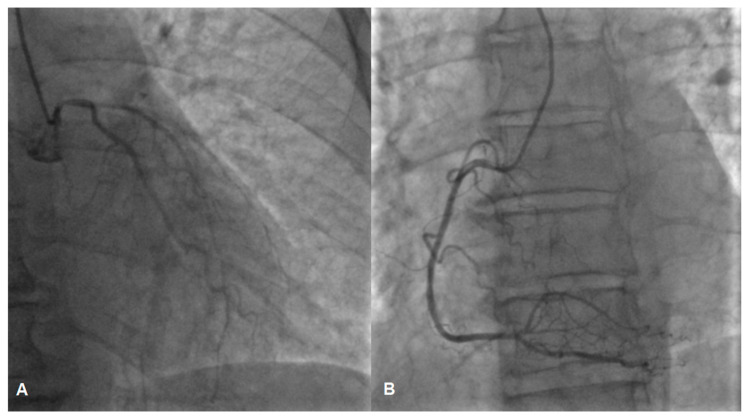
Vasospasm of the left (**A**) and the right (**B**) coronary artery, possible lesion of the left main (**A**).

**Figure 3:**
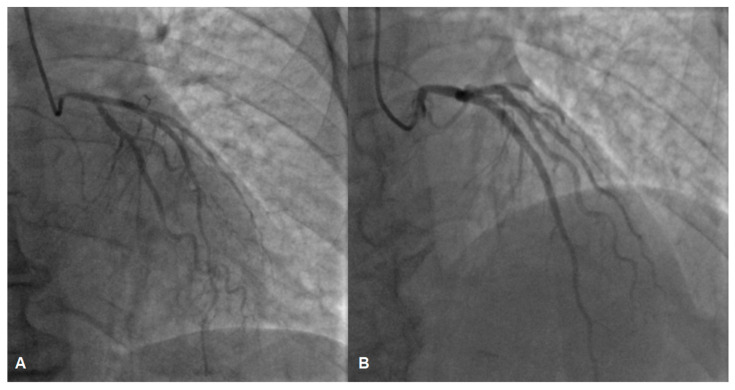
The left coronary artery before (**A**) and after (**B**) intracoronary nitroglycerin injection, angiography depicts partial relief of vasospasm, suspicion of a lesion in the left main coronary artery (**B**).

While awaiting coronary angiography, the patient experienced a recurrence of chest pain, prompting an urgent procedure. Subsequent coronary angiography revealed constrictions in all coronary arteries, including the left main coronary artery (Figure 2A,B). Suspecting diffuse vasospasm, intracoronary nitroglycerin was administered. Following the injection of 400 mcg of intracoronary nitroglycerin, partial relief of the diffuse vasospasm was observed, although the proximal segment of the left main artery remained affected (Figure 3A,B).

**Figure 4:**
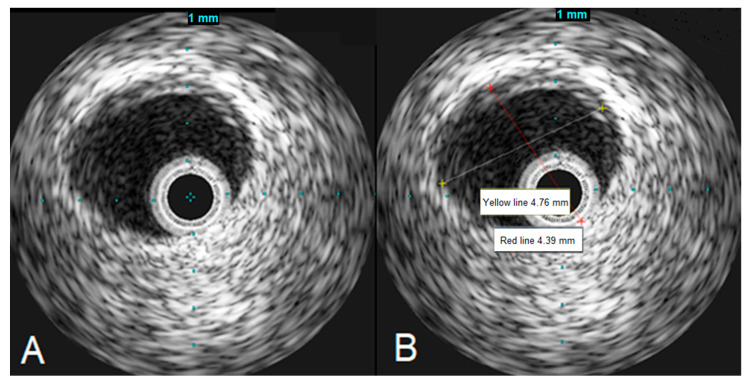
IVUS images demonstrating the left main artery without significant atherosclerosis (**A**), the left main artery diameter appears to be normal (**B**).

**Figure 5:**
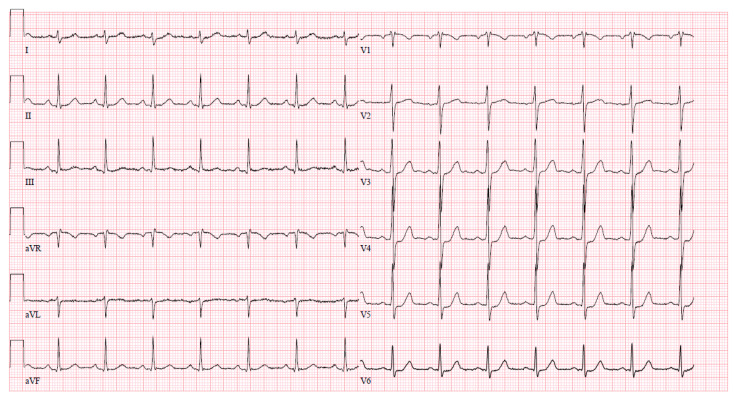
Normal ECG immediately after coronary angiography.

To assess the left main stenosis, intravascular ultrasound (IVUS) was employed. IVUS revealed the absence of significant atherosclerotic lesion in the left main, confirming the diagnosis of vasospasm (Figure 4). The other sites of the left coronary artery appeared to be without any lesions as well, except the proximal part of LAD, where a minor plaque was found during the IVUS examination. After the procedure, serial ECGs did not demonstrate any ischemic changes (Figure 5).

**Figure 6:**
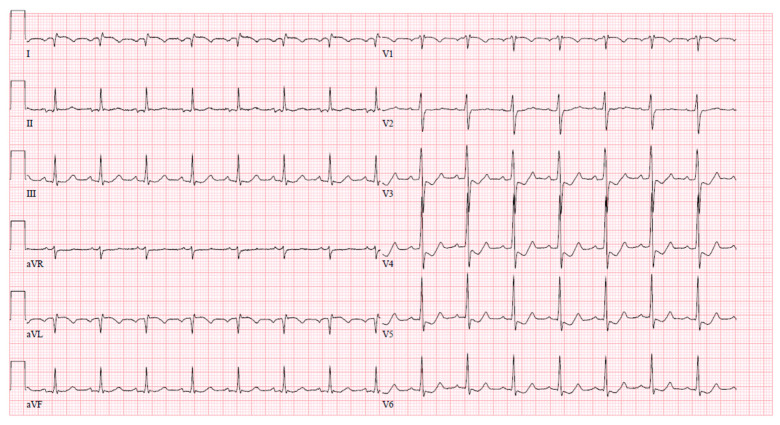
Ischemic ECG changes in cardiac ICU after coronary angiography.

After coronary angiography, at the cardiac ICU, the patient experienced another wave of chest pain with ischemic changes observed on the ECG (ST elevation with negative T waves in leads I and aVL, ST depression in V3–6) (Figure 6). The episode was successfully managed with intravenous nitrates.

Biochemical blood tests revealed thyrotoxicosis, TSH was 0.000 mU/L, free thyroxine (T4) was elevated at 40.19 pmol/L (normal range 9.0–19.0 pmol/L), and free triiodothyronine (T3) was above detectable concentration > 30.72 pmol/L (normal range 2.43–6.01 pmol/L). All blood tests during the hospital stay are provided in Table 3.

The patient was consulted by an endocrinologist; recommended therapy was methimazole, initial dosage 10 mg 3 times daily. After 7–10 days, the dose could be gradually reduced by 5 mg, eventually reaching 5–10 mg daily maintenance dose. Eventually, the patient was discharged symptoms-free, expecting good prognosis on conservative management. All prescribed long-term medications on discharge are shown in Table 4.

The patient was contacted six months after discharge from the PCI center. The patient is currently taking prescribed medications, including methimazole 5 mg daily for thyrotoxicosis. Since discharge, the patient has not reported any chest pain or heart-related health issues.

## 3. Discussion

The presented case highlights a rare occurrence of acute thyrotoxicosis precipitating unstable vasospastic angina in a middle-aged female patient without underlying coronary artery pathology. Similar occurrences have been documented in previous reports. Coronary vasospasm can often mimic acute coronary syndrome stemming from an atherosclerotic event, exhibiting typical ECG changes, and sometimes even elevated high sensitivity troponin levels, coupled with persistent chest pain, thus posing challenges in clinical differentiation prior to the coronary angiography [9,10]. Also, vasospasm can clinically resemble ST-elevation myocardial infarction, manifesting with consistent ECG findings and elevated troponin levels [11,12]. Therefore, during coronary angiography, the administration of intracoronary nitroglycerin serves as a valuable diagnostic tool in distinguishing vasospasm from atherosclerotic coronary artery disease and avoiding unnecessary stenting [9,10,11]. Thyrotoxicosis-induced coronary vasospasm may lead to severe outcomes, including cardiac arrest and necessitating resuscitation, as reported by Omar et al. [13]. Moreover, a patient experiencing a thyroid storm has been reported to develop spontaneous coronary artery dissection (SCAD), which is another causative factor of ACS to remember. This condition composes up to 4% of ACS causes [14]. However, the current literature lacks sufficient evidence to establish a causative association between thyroid status and SCAD. Previously documented instances underscore the potential for life-threatening clinical scenarios arising from thyrotoxicosis-induced acute coronary syndromes. 

The thyroid hormone profile tends to have a controversial impact on the incidence and prognosis of ACS across the literature. For instance, Pimentel et al. suggest that euthyroid sick syndrome (ESS) indicates a poorer overall prognosis for ACS patients [15], although ESS is likely a consequence of ACS rather than a cause [16]. A similar conclusion was reached by Lamprou et al. [17]. While some studies conclude that TSH levels and thyroid status have no impact on ACS outcomes [18], there is a body of growing evidence that thyroid pathology exacerbates ACS outcomes [19,20]. Atypical thyroid status worsens ventricular function, elevates cholesterol levels, and impacts heart rate and rhythm; thus, it might deteriorate the prognosis of an ACS event [20]. Hyperthyroidism affects the rate and prognosis of ACS primarily by worsening ventricular function, inducing tachyarrhythmias and provoking vasospasm [20]. Hypothyroidism is associated with hypercholesterolemia, thereby increasing the likelihood of coronary heart disease and ACS [21,22]. Moreover, subclinical hypothyroidism is linked to abnormal global longitudinal strain in patients with heart failure, which is a common consequence of ACS [23]. Thus, hypothyroidism may also worsen outcomes for ACS patients and requires precise monitoring and treatment [22,23]. Nevertheless, future research is crucial to provide stronger evidence. Given all the evidence, we would recommend TSH screening in ACS patients, especially if there is a clinical suspicion of the vasospasm-induced ACS. 

Based on a comparative study by Lee et al., coronary vasospasm-induced ACS appears to be more prevalent among patients with thyrotoxicosis compared to those who are euthyroid [24]. These patients typically exhibit coronary arteries without angiographically detectable lesions [24]. Additionally, spontaneous coronary artery spasm tends to be more frequent in association with thyrotoxicosis, while euthyroid patients experience coronary vasospasm primarily during provocation tests [24]. Moreover, diffuse spasm, the left main artery vasospasm, and intractable spasm are more commonly observed in cases of thyrotoxicosis [24]. The disease is more prevalent in middle-aged or elderly female population [25], and this pattern aligns with our presented case. Furthermore, coronary vasospasm in thyrotoxicosis demonstrates a weak association with traditional cardiovascular risk factors such as dyslipidemia, smoking, and hypertension, thereby suggesting thyrotoxicosis as an independent risk factor [24]. However, it is worth noting that coronary vasospasm tends to occur more frequently in patients with concomitant stenosing coronary atherosclerosis [26]. Collectively, according to these findings, thyrotoxicosis may be an independent risk factor for coronary vasospasm, particularly in the female population. 

Thyrotoxicosis shortens myocardial refractory time and induces sinus tachycardia, thereby increasing oxygen demand alongside coronary vasospasm [24,27]. This leads to inadequate oxygen supply to cardiomyocytes, serving as a pivotal mechanism behind thyrotoxicosis-induced cardiovascular events, particularly myocardial infarction [24,27,28]. Furthermore, both T4 and T3 hormones enhance adrenergic activity even in the presence of normal blood catecholamine levels [29,30]. Consequently, thyroid hormones augment vascular sensitivity to catecholamines, triggering smooth muscle contraction and inducing vasospasm [31]. Another contributing factor to vasospasm is the thyrotoxicosis-induced oxidative stress in the smooth muscle layer of the arteries, which amplifies vessel tone [32]. These factors collectively render coronary arteries hypersensitive to various stimuli [31,32]. Notably, smoking has been identified as a contributing factor to coronary artery vasospasm in the context of thyrotoxicosis with studies suggesting that vasospasm tends to be more severe among smokers [33,34]. Additionally, thyrotoxicosis exerts a notable impact on the coagulation system, instigating a prothrombotic state characterized by increased coagulation and reduced fibrinolysis activity [35]. This results from decreased concentrations of antithrombotic factors such as protein C and elevated levels of prothrombotic compounds [28,34]. Thyrotoxicosis is a common clinical picture of Grave’s disease which involves autoimmune mechanisms that elevate levels of interleukin-6 (IL-6), which is a cytokine contributing to the autoimmune storm [36]. Importantly, IL-6 levels tend to rise during acute coronary syndromes [37]. Therefore, the resulting proinflammatory state during thyrotoxicosis may play a significant role in the development of cardiovascular events. In summary, the main contributing factors of thyrotoxicosis to acute coronary syndromes encompass inadequate oxygen supply, heightened vascular contractility and sensitivity, and the proinflammatory and hypercoagulable states. 

Another important topic to discuss is aspirin use in coronary vasospasm patients. The literature presents a mild dispute: for example, Lee Y et al. found that aspirin may have a preventive effect on ACS recurrence in patients with coronary vasospasm without significant coronary lesions [38]. However, a recent systematic review and meta-analysis by Lin et al. of four propensity matched cohorts comprising 3661 patients concluded that aspirin use is not beneficial in terms of reducing future ACS events in patients with coronary vasospasm without significant coronary atherosclerosis [39]. Moreover, it is known that aspirin inhibits COX-2 action and the production of prostaglandins and prostacyclins, which serve as vasodilating agents and promote angiogenesis [40,41]. Therefore, aspirin may exacerbate symptoms related to coronary vasospasm. Thus, based on recent literature, aspirin should not be routinely prescribed for similar patients unless other indications are present. 

To treat and prevent vasospasm attacks, the patient was prescribed diltiazem, and long-acting nitrate (ISDN) and did not reach the normal thyroid state upon discharge. Currently, there is insufficient evidence regarding the prescription of antianginal treatment for thyrotoxicosis-induced coronary vasospasm, and no articles were found that discuss whether it should be continued once the patient becomes euthyroid. We would suggest continuing antianginal treatment until thyroid storm is controlled and the euthyroid state is reached. Since antianginal drugs provide symptomatic treatment, they may be discontinued once the risk factor for coronary vasospasm is eliminated. However, since some of the medications used to treat vasospasm also act as a blood pressure-lowering agent, they might be continued long term to maintaining normal blood pressure and to preventing vasospasm for patients with arterial hypertension.

In the literature, intracoronary nitroglycerin is consistently recognized as a vital diagnostic tool during coronary angiography especially when coronary vasospasm is suspected [9,10,11,12,24]. Moreover, IVUS plays a crucial role in optimizing the diagnostic process and averting unnecessary stenting particularly in cases of refractory coronary artery spasm resistant to nitroglycerin [12,24,42]. Therefore, when feasible, it is advisable to utilize IVUS prior to PCI when the cause of coronary stenoses remains unclear [43]. However, urgent revascularization, encompassing PCI and coronary artery bypass grafting (CABG), may be essential for salvaging lives in scenarios of refractory coronary vasospasm [44]. This urgency is particularly evident when intractable vasospasm induces significant spasm in the left main coronary artery, resulting in diffuse myocardial injury and potentially life-threatening arrhythmias [43]. According to the existing literature, the outcomes of acute coronary syndromes induced by coronary vasospasm do not significantly differ between patients with thyrotoxicosis and those who are euthyroid [24]. Nevertheless, achieving an euthyroid state is widely acknowledged to substantially improve long-term outcomes among thyrotoxicosis patients, alleviating angina symptoms and promoting a favorable prognosis [25,44,45]. In summary, the recent literature underlines the paramount importance of intracoronary nitroglycerin and IVUS in cases of coronary vasospasm and other ambiguous scenarios. Incorporating these techniques into clinical practice can help mitigate the incidence of unnecessary PCI and stenting, ultimately leading to improved overall patient outcomes.

## 4. Conclusions

Clinically significant coronary vasospasm triggered by thyrotoxicosis remains a rarity in clinical practice, often posing diagnostic challenges. Misdiagnosing coronary artery spasm as an atherosclerotic lesion often leads to unnecessary stenting. The presented case emphasizes the critical role of intracoronary nitroglycerin and IVUS in distinguishing the underlying cause of angiographic coronary lesions especially in complex cases where the patient’s coronary history is either negative or unknown. We advocate for the utilization of these techniques to optimize invasive coronary artery diagnostics, enabling the selection of the most appropriate treatment strategies and ultimately improving long-term prognosis.

## Figures and Tables

**Table 1:** Prescribed medications in the secondary care hospital.

Drug, Form	Dosage, Regime
Furosemide i/v	60 mg every second day
Nadroparin s/c	0.6 mL b.i.d.
Torasemide p/os	20 mg every second day
Aspirin p/os	100 mg o.d.
Verapamil and Trandolapril p/os	180/2 mg p/os o.d.
Isosorbide mononitrate (ISMN) p/os	20 mg o.d.

**Table 2:** Prescribed medications in the PCI center.

Drug, Form	Dosage, Regime
Nadroparin s/c before coronary angiography	0.6 mL b.i.d.
Aspirin p/os	100 mg o.d.
Pantoprazole p/os	40 mg o.d.
Metoprolol p/os (later changed to Diltiazem)	47.5 mg o.d.
Methimazole p/os (after endocrinologist consult)	5 mg t.i.d.
Isosorbide dinitrate (ISDN) p/od	20 mg o.d.
Molsidomine p/o	4 mg o.d.
Diltiazem p/os	90 mg b.i.d.

**Table 3:** Laboratory tests.

Laboratory Test	Reference Value	Initial	Day 4	Day 6
Hemoglobin (g/L)	120–156	121	-	**114**
White blood cells (×10^9^/L)	3.6–10.5	3.73	-	4.26
Platelets (×10^9^/L)	150–370	250	-	202
Thyroid hormones				
Thyroid-stimulating hormone (mU/L)	0.4–4.0	**0.000**	**0.001**	**-**
Triiodothyronine (pmol/L)	2.43–6.01	**>30.72**	-	-
Thyroxine (pmol/L)	9.0–19.0	**40.19**	**28.72**	-
General biochemical blood test				
C-reactive protein (mg/L)	≤5	1.00	-	-
Glucose (mmol/L)	4.2–6.1	6.09	-	-
Troponin I (ng/L)	≤16	12	-	-
Creatinine (µmol/L)	49–90	**41**	-	-
Electrolytes				
Potassium (mmol/L)	3.8–5.3	4.4	-	-
Sodium (mmol/L)	134–145	142	-	-
Chloride (mmol/L)	98–107	107	-	-
Liver enzymes				
Aspartate transaminase (U/L)	≤40	57	-	23
Alanine transaminase (U/L)	≤40	56	-	31
Lipid profile				
Total cholesterol (mmol/L)	<5.2	3.17	-	-
LDL cholesterol (mmol/L)	<1.4	**1.52**	-	-
HDL cholesterol (mmol/L)	>1.2	**1.08**	-	-
Non-HDL cholesterol (mmol/L)	<2.2	2.09	-	-
Triglycerides (mmol/L)	≤1.7	1.24	-	-

LDL—low-density lipoprotein, HDL—high-density lipoprotein.

**Table 4:** Prescribed long-term medications.

Drug, Form	Dosage, Regime
Aspirin p/os	100 mg o.d.
Omeprazole p/os	20 mg o.d.
Ivabradine p/os	7.5 mg b.i.d.
Methimazole p/os	5 mg q.i.d.
Isosorbide dinitrate (ISDN) p/os	20 mg o.d.
Molsidomine p/os	4 mg o.d.
Diltiazem p/os	90 mg b.i.d.

## Data Availability

Not applicable.

## References

[1] Byrne R.A., Rossello X., Coughlan J.J., Barbato E., Berry C., Chieffo A., Claeys M.J., Dan G.-A., Dweck M.R., Galbraith M. (2023). 2023 ESC Guidelines for the management of acute coronary syndromes: Developed by the task force on the management of acute coronary syndromes of the European Society of Cardiology (ESC). Eur. Heart J..

[2] Singh A., Museedi A.S., Grossman S.A. (2023). Acute Coronary Syndrome. StatPearls [Internet].

[3] Helwani M.A., Amin A., Lavigne P., Rao S., Oesterreich S., Samaha E., Brown J.C., Nagele P. (2018). Etiology of Acute Coronary Syndrome after Noncardiac Surgery. Anesthesiology.

[4] Kim H.J., Jo S.H., Lee M.H., Seo W.W., Baek S.H. (2020). Hyperthyroidism Is Associated with the Development of Vasospastic Angina, but Not with Cardiovascular Outcomes. J. Clin. Med..

[5] Swarup S., Patibandla S., Grossman S.A. (2023). Coronary Artery Vasospasm. StatPearls [Internet].

[6] Hung M.J., Hsu K.H., Hu W.S., Chang N.C., Hung M.Y. (2013). C-reactive protein for predicting prognosis and its gender-specific associations with diabetes mellitus and hypertension in the development of coronary artery spasm. PLoS ONE.

[7] Hung M.Y., Hsu K.H., Hung M.J., Cheng C.W., Kuo L.T., Cherng W.J. (2009). Interaction between cigarette smoking and high-sensitivity C-reactive protein in the development of coronary vasospasm in patients without hemodynamically significant coronary artery disease. Am. J. Med. Sci..

[8] Blick C., Nguyen M., Jialal I. (2023). Thyrotoxicosis. StatPearls [Internet].

[9] Anjum R., Virk H.U.H., Goyfman M., Lee A., John G. (2022). Thyrotoxicosis-Related Left Main Coronary Artery Spasm Presenting as Acute Coronary Syndrome. Cureus.

[10] Klomp M., Siegelaar S.E., van de Hoef T.P., Beijk M.A.M. (2020). A case report of myocardial infarction with non-obstructive coronary artery disease: Graves’ disease-induced coronary artery vasospasm. Eur. Heart J. Case Rep..

[11] Rymer De Marchena I., Gutman A., Zaidan J., Yacoub H., Hoyek W. (2017). Thyrotoxicosis Mimicking ST Elevation Myocardial Infarction. Cureus.

[12] Diečkus L., Rodevič G., Baranauskas A., Davidavičius G., Budrys P. (2023). Case report: A rare manifestation of vasospasm induced myocardial infarction with ST-segment elevation in a young male patient. Front. Cardiovasc. Med..

[13] Omar A.M.A., Knott K., Saba M.M., Lim P.O. (2023). Cardiac arrest in myocardial infarction with non-obstructive coronary artery (MINOCA) secondary to thyroid dysfunction. BMJ Case Rep..

[14] Muscoli S., Lecis D., Prandi F.R., Ylli D., Chiocchi M., Cammalleri V., Lauro D., Andreadi A. (2022). Risk of sudden cardiac death in a case of spontaneous coronary artery dissection presenting with thyroid storm. Eur. Rev. Med. Pharmacol. Sci..

[15] Pimentel R.C., Cardoso G.P., Escosteguy C.C., Abreu L.M. (2006). Thyroid hormone profile in acute coronary syndromes. Arq. Bras. Cardiol..

[16] McDermott M.T., Parsons P.E., Wiener-Kronish J.P. (2007). Chapter 52—Thyroid Disease in the Intensive Care Unit. Critical Care Secrets.

[17] Lamprou V., Varvarousis D., Polytarchou K., Varvarousi G., Xanthos T. (2017). The role of thyroid hormones in acute coronary syndromes: Prognostic value of alterations in thyroid hormones. Clin. Cardiol..

[18] Abdulaziz Qari F. (2015). Thyroid Hormone Profile in Patients with Acute Coronary Syndrome. Iran. Red. Crescent Med. J..

[19] Gürdoğan M., Altay S., Korkmaz S., Kaya Ç., Zeybey U., Ebik M., Demir M. (2019). The Effect of Thyroid Stimulating Hormone Level within the Reference Range on In-Hospital and Short-Term Prognosis in Acute Coronary Syndrome Patients. Medicina.

[20] Kumar R., Sinha R., Gunjan G., Singh S.K. (2024). A Cross-Sectional Study of Acute Coronary Syndrome and Thyroid Profile: Dissecting the Relationship to Improve Patient Care. Cureus.

[21] Dhital R., Basnet S., Poudel D.R. (2017). Impact of Hypothyroidism on Occurrence and Outcome of Acute Coronary Syndrome from the National Inpatient Sample. Am. J. Cardiol..

[22] Han C., Xu K., Wang L., Zhang Y., Zhang R., Wei A., Dong L., Hu Y., Xu J., Li W. (2022). Impact of persistent subclinical hypothyroidism on clinical outcomes in non-ST-segment elevation acute coronary syndrome undergoing percutaneous coronary intervention. Clin. Endocrinol..

[23] Javed N., Hayagreev V., DeLaCruz A., Saad M., Singh A., Vittorio T. (2023). The Role of Global Longitudinal Strain in Subclinical Hypothyroid Patients with Heart Failure. Cureus.

[24] Lee S.Y., Yu C.W., Choi Y.J., Choi R.K., Park J.S., Lee H.J., Kim J.S., Jang H.J., Jang D.H., Chae M.J. (2014). A comparison of clinical features of coronary artery spasm with and without thyrotoxicosis. Coron. Artery Dis..

[25] Choi Y.H., Chung J.H., Bae S.W., Lee W.H., Jeong E.M., Kang M.G., Kim B.G., Kim K.W., Park G.E. (2005). Severe coronary artery spasm can be associated with hyperthyroidism. Coron. Artery Dis..

[26] JCS Joint Working Group (2010). Guidelines for diagnosis and treatment of patients with vasospastic angina (coronary spastic angina) (JCS 2008): Digest version. Circ. J..

[27] Quiroz-Aldave J.E., Durand-Vásquez M.d.C., Lobato-Jeri C.J., Muñoz-Moreno J.-M., Condori D.C.D.G., Ildefonso-Najarro S.P., Contreras-Yametti F., Zavaleta-Gutiérrez F., Concepción-Urteaga L., Concepción-Zavaleta M.J. (2023). Thyrotoxic Cardiomyopathy: State of the Art. Eur. Endocrinol..

[28] Kim H.J., Jung T.S., Hahm J.R., Hwang S.-J., Lee S.M., Jung J.H., Kim S.K., Chung S.I.I. (2011). Thyrotoxicosis-induced acute myocardial infarction due to painless thyroiditis. Thyroid.

[29] Nayak B., Burman K. (2006). Thyrotoxicosis and thyroid storm. Endocrinol. Metab. Clin. N. Am..

[30] Chang K.H., Chang W.C., Su C.S., Liu T.J., Lee W.L., Lai C.H. (2017). Vasospastic myocardial infarction complicated with ventricular tachycardia in a patient with hyperthyroidism. Int. J. Cardiol..

[31] McAllister R.M., Grossenburg V.D., Delp M.D., Laughlin M.H. (1998). Effects of hyperthyroidism on vascular contractile and relaxation responses. Am. J. Physiol..

[32] Zwaveling J., Prins E.A., Maas M.A., Pfaffendorf M., Van Zwieten P.A. (1996). The influence of hyperthyroidism on pharmacologically induced contractions of isolated resistance arteries. Eur. J. Pharmacol..

[33] Lewandowski K.C., Rechciński T., Krzemińska-Pakuła M., Lewiński A. (2010). Acute myocardial infarction as the first presentation of thyrotoxicosis in a 31-year old woman—Case report. Thyroid. Res..

[34] Ashikaga T., Nishizaki M., Fujii H., Niki S., Maeda S., Yamawake N., Kishi Y., Isobe M. (2007). Examination of the microcirculation damage in smokers versus nonsmokers with vasospastic angina pectoris. Am. J. Cardiol..

[35] Elbers L.P.B., Fliers E., Cannegieter S.C. (2018). The influence of thyroid function on the coagulation system and its clinical consequences. J. Thromb. Haemost..

[36] Lee H.J., Lombardi A., Stefan M., Li C.W., Iii W.B.I., Owen R.P., Concepcion E., Tomer Y. (2017). CD40 Signaling in Graves Disease Is Mediated Through Canonical and Noncanonical Thyroidal Nuclear Factor κB Activation. Endocrinology.

[37] Alevizos M., Karagkouni A., Panagiotidou S., Vasiadi M., Theoharides T.C. (2014). Stress triggers coronary mast cells leading to cardiac events. Ann. Allergy Asthma Immunol..

[38] Lee Y., Park H.C., Shin J. (2018). Clinical efficacy of aspirin with identification of intimal morphology by optical coherence tomography in preventing event recurrence in patients with vasospasm-induced acute coronary syndrome. Int. J. Cardiovasc. Imaging.

[39] Lin Y., Chen Y., Yuan J., Qin H., Dong S., Chen Q. (2021). Impact of aspirin use on clinical outcomes in patients with vasospastic angina: A systematic review and meta-analysis. BMJ Open.

[40] Vane J.R., Botting R.M. (2003). The mechanism of action of aspirin. Thromb. Res..

[41] Ozen G., Norel X. (2017). Prostanoids in the pathophysiology of human coronary artery. Prostaglandins Other Lipid Mediat..

[42] Khan A., Lahmar A., Riasat M., Ehtesham M., Asif H., Khan W., Haseeb M., Boricha H. (2022). Myocardial Infarction with Non-obstructive Coronary Arteries: An Updated Overview of Pathophysiology, Diagnosis, and Management. Cureus.

[43] Park Y.M., Kang W.C., Shin K.C., Han S.H., Ahn T., Choi I.S., Shin E.K. (2012). Repeated sudden cardiac death in coronary spasm: Is IVUS helpful to decide treatment strategy?. Int. J. Cardiol..

[44] Al Jaber J., Haque S., Noor H., Ibrahim B., Al Suwaidi J. (2010). Thyrotoxicosis and coronary artery spasm: Case report and review of the literature. Angiology.

[45] Lee S.M., Jung T.S., Hahm J.R., Im S.I., Kim S.K., Lee K.J., Lee J.M., Chun S.I. (2007). Thyrotoxicosis with coronary spasm that required coronary artery bypass surgery. Intern. Med..

